# Niche width collapse in a resilient top predator following ecosystem fragmentation

**DOI:** 10.1111/j.1461-0248.2007.01087.x

**Published:** 2007-10

**Authors:** Craig A Layman, John P Quattrochi, Caroline M Peyer, Jacob E Allgeier, Katharine Suding

**Affiliations:** 1 Marine Sciences Program, Department of Biological Sciences, Florida International University 3000 NE 151st St, North Miami, FL 33181, USA; 2 Institute of Ecology, University of Georgia Athens, GA 30602, USA

**Keywords:** Biodiversity, ecosystem function, Elton, food chain length, food web, functional diversity, hydrologic connectivity, individual specialization, optimal foraging theory, stable isotope analysis

## Abstract

Much research has focused on identifying species that are susceptible to extinction following ecosystem fragmentation, yet even those species that persist in fragmented habitats may have fundamentally different ecological roles than conspecifics in unimpacted areas. Shifts in trophic role induced by fragmentation, especially of abundant top predators, could have transcendent impacts on food web architecture and stability, as well as ecosystem function. Here we use a novel measure of trophic niche width, based on stable isotope ratios, to assess effects of aquatic ecosystem fragmentation on trophic ecology of a resilient, dominant, top predator. We demonstrate collapse in trophic niche width of the predator in fragmented systems, a phenomenon related to significant reductions in diversity of potential prey taxa. Collapsed niche width reflects a homogenization of energy flow pathways to top predators, likely serving to destabilize remnant food webs and render apparently resilient top predators more susceptible to extinction through time.

## Introduction

Organisms differ in their susceptibility to ecosystem fragmentation and other anthropogenic impacts (McKinney 1997; Henle *et al.* 2004; Cardillo *et al.* 2005). For example, trophic generalists may be less susceptible to extinction (or extirpation) because of their ability to shift among alternative food resources (Purvis *et al.* 2000; Hopkins *et al.* 2002). As such, those organisms with a broad trophic niche may be expected, other factors being equal, to be most resilient to the detrimental impacts associated with fragmentation. But as species loss at local and global scales continues (Thomas *et al.* 2004; Pounds *et al.* 2006), there is a pressing need to not only examine which species are most susceptible to effects stemming from ecosystem fragmentation, but also to understand the ecology of those resilient species that are able to persist in altered habitats. Specifically, is the trophic ecology of resilient top predators altered in fragmented habitats? If so, how is this related to overall food web architecture and what are implications for ecosystem function and stability?

The trophic niche is one of the longest-standing approaches to the study of organisms’ dietary ecology (Elton 1927). Charles Elton defined a species’‘niche’ as the sum of all the interactions (especially trophic) that link it to other species in an ecosystem (Elton 1927), i.e. the specified trophic ‘role’ of that species (Leibold 1995). Niche width (or breadth) traditionally has been quantified using direct stomach content analysis, in conjunction with measures of richness and evenness applied across individuals from a population (Bearhop *et al.* 2004). However, due to the well-known limitations of these approaches, stable isotopes have become a common alternative for study of trophic niches, providing for time- and space-integrated representations of the trophic ecology of organisms (Post *et al.* 2000b; Bearhop *et al.* 2004; Layman *et al.* 2007). For example, ratios of ^15^N to ^14^N (expressed as δ^15^N) exhibit stepwise enrichment with trophic transfers, and thus can be used to estimate an organism’s trophic position. Ratios of carbon isotopes (δ^13^C) vary substantially among primary producers with different photosynthetic pathways (e.g. C3 vs. C4 plants), but change little with trophic transfers, and thus can be used to determine ultimate sources of dietary carbon (Post 2002b; Bearhop *et al.* 2004). As such, relative position of organisms in δ^13^C–δ^15^N bi-plot space (a 2-D ‘niche space’) can reveal important aspects of trophic structure, and novel quantitative metrics based on these representations of the niche may be powerful tools to test ecological theory and study ecological responses to anthropogenic impacts (Layman *et al.* 2007; Schmidt *et al.* 2007).

Ecosystem fragmentation is one of the core drivers of biodiversity loss at local, regional and global scales (Fahrig 2003). Conceptual and theoretical discussions of fragmentation have been dominated by a focus on terrestrial ecosystems (Robinson *et al.* 1995; Debinski & Holt 2000; Fahrig 2003), yet aquatic ecosystem fragmentation is also widespread and can significantly reduce biodiversity and alter ecosystem function (Layman *et al.* 2004; Nilsson *et al.* 2005; Greathouse *et al.* 2006; Pringle 2006). Aquatic ecosystem fragmentation is defined as the disruption of hydrologic connectivity, i.e. disruption of the water-mediated transfer of matter, energy, or organisms within or between elements of the hydrologic cycle (Pringle 2001). Most well-studied instances of aquatic ecosystem fragmentation relate to dams that disrupt connectivity in freshwater rivers or streams, thereby intrinsically altering ecological processes and ecosystem function (Nilsson *et al.* 2005; Greathouse *et al.* 2006; Pringle 2006). We focus on anthropogenic fragmentation of estuarine tidal creeks (Layman *et al.* 2004; Valentine *et al.* 2007a, b), thereby expanding the study of fragmentation to one of the most severely impacted types of ecosystems at a global scale (Lotze *et al.* 2006).

We propose a novel stable isotope approach, based on relative position of individuals in δ^13^C–δ^15^N niche space, to directly quantify trophic niche width. We demonstrate that trophic niche width of a dominant top predator collapses following aquatic ecosystem fragmentation, and we discuss how this collapse may further decrease food web stability in fragmented habitats.

## Methods

### Study sites

Our study systems were clearwater, mangrove-lined tidal creeks located on Abaco Island, Bahamas. These estuarine creeks are critically important in the Bahamas, both ecologically and economically, because of their role as nursery habitats and feeding areas, and for the valuable ecosystem services they provide (Buchan 2000). These systems are generally characterized by a relatively narrow mouth (lined by red mangrove, *Rhizophora mangle*) that opens to a broad, shallow wetland area. Substrate is variable, and includes fine silt, coarse sand, rocky outcroppings, seagrass and macroalgae. Faunal and floral diversity is high, and is dependent on the degree of hydrologic connectivity to the marine environment (Layman *et al.* 2004; Valentine *et al.* 2007a, b).

Tidal creeks in the Bahamas range in size from several hectares with maximum low tide depths of 1 m, to thousands of hectares with maximum depths > 10 m. Our collections were made in systems that fall at the lower end of this range. The relationship between ecosystem (i.e. creek) size and niche width was not examined directly herein because of the difficulty in explicitly determining the ‘size’ of such highly-connected (i.e. to the adjacent marine environment) and dynamic ecosystems. But because fragmentation and creek size may be negatively correlated in some instances (see Discussion), we specifically chose creeks that would yield an orthogonal gradient between creek size and fragmentation. For example, the largest systems were among some of the most– (Lake City) and least– (Cross Harbour) fragmented.

Fragmentation of creeks results from construction of roads, typically near the narrow creek mouth, that lack flow conveyance structures (e.g. bridge or culverts). Creek sites were chosen to reflect a complete gradient of fragmentation, from creeks with no tidal exchange to those with a mean daily tidal range of *c.* 0.8 m. Degree of fragmentation was estimated using HOBO® water level loggers, (Onset, Pocasset, MA, USA) at each collection site. Over a 2-week period, the mean daily tidal amplitude (i.e. daily maximum water depth – daily minimum water depth) was calculated at the site in a creek where snapper were collected. Mean ocean tidal amplitude (i.e. daily ocean maximum water depth – daily ocean minimum water depth) over the same time period was used as a measure of the maximum possible creek amplitude. ‘Percent fragmentation’ was determined based on the reduction in mean daily tidal amplitude at the creek site relative to the maximum possible amplitude, and was employed as the core independent variable in statistical analysis. This variable is a direct proxy of the amount of tidal exchange, and thus the overall degree of hydrologic connectivity between interior wetlands and the marine environment. Many variables influence the degree of connectivity (e.g. distance of collection site from the ocean and creek bathymetry), but tidal amplitude is a composite variable that encapsulates all factors that affect overall connectivity with the marine environment. In this sense, we employ a *structural* measure of connectivity (*sensu*Crooks & Sanjayan 2006), i.e. a proxy for the total amount of water exchanged between high and low tide on a daily basis.

Our study species was the grey snapper (*Lutjanus griseus* Linneaus), an ecologically and economically critical fish species (Starck & Schroeder 1971), that is one of the dominant species (in terms of abundance, biomass and production) in Bahamian tidal creeks regardless of the degree of fragmentation (Layman *et al.* 2004; Valentine *et al.* 2007a). This species is regarded as a generalist predator that consumes a wide variety of prey items (Layman & Silliman 2002; Cocheret de la Morinière *et al.* 2003). We constrained the size of individuals collected to 120–225 mm to control for ontogenetic diet shifts. This size range represents 2- to 4-year-old individuals in these creek systems (A. L. Rypel, unpublished data). We collected 13 individuals from each of 11 creek systems. Sample size was determined based on preliminary analysis of the relationship between number of individuals and values of the niche width metric in creek sites with high among-individual niche variation (unfragmented sites). Captured fish were immediately euthanized by pithing and *c.* 2 g of muscle tissue was removed just below the dorsal fin. Sample analysis follows Post *et al.* (2007) and was conducted at the Yale Earth System Center for Stable Isotopic Studies (ESCSIS). All stable-isotope values are reported in the δ notation where δ^13^C or δ^15^N = [(*R*_sample_/*R*_standard_) − 1] × 1000, where *R* is ^13^C/^12^C or ^15^N/^14^N.

Individuals from each of the 11 populations were graphed in δ^13^C–δ^15^N niche space, and quantitative metrics were calculated independently for each population. Such metrics can reveal important aspects of intraspecific trophic structure, and are powerful tools useful for studying ecological responses to anthropogenic impacts (Layman *et al.* 2007; Schmidt *et al.* 2007). The measure of niche width employed is based on the area of the convex hull (TA) bounding a sub-set of individuals of a population. TA is calculated as the total area encompassed by the smallest convex polygon containing these individuals in δ^13^C and δ^15^N niche space. TA is a direct measure of population niche width, as it is a composite metric that reflects variation along both δ^13^C and δ^15^N niche dimensions (Layman *et al.* 2007). Convex hull areas were calculated using Arc View GIS 3.3 (ESRI, Redlands, CA, USA). Additional information on application of convex hulls in ecological studies can be found in Cornwell *et al.* (2006) and Layman *et al.* (2007).

To examine abundance of potential prey items in creeks across the fragmentation gradient, we compiled a metadatabase of all taxa we have identified in analyses of > 400 individual snapper’s stomach contents. Subsequently, we exhaustively sampled creeks for each of these taxa using a variety of methodologies, including traps, seine nets, cast nets, dip nets, breaking rocks and sieving contents of algal mats. We used a familial level of taxonomic identification because this was the level to which the majority of prey items in snapper stomachs could be identified. Based on published natural history and trophic ecology of these taxa, we classified each potential prey family into their most appropriate functional grouping (largely based on trophic ecology) following an approach similar to Micheli & Halpern (2005). Functional groups also were established at the familial level to remain consistent with stomach content analysis classifications.

To further explore trophic architecture in representative creek systems, we expanded our sampling to collect all organisms ‘resident’ (i.e. do not move out of the creeks at low tide) to tidal creeks. That is, we collected not only potential snapper prey, but also all other resident fishes (e.g. grunts, parrotfish) and invertebrates (e.g. snails, sponges) present. Muscle tissue was processed for fishes and large invertebrates, and the whole organism for smaller invertebrates. Separate samples were analysed for δ^13^C and δ^15^N for invertebrate taxa, one of which was acidified to remove inorganic carbon before δ^13^C analysis. Two to 13 individuals were processed for each of the species, and the species mean values were plotted in δ^13^C–δ^15^N niche space for community-level examination following Layman *et al.* (2007).

## Results

**Figure 1:**
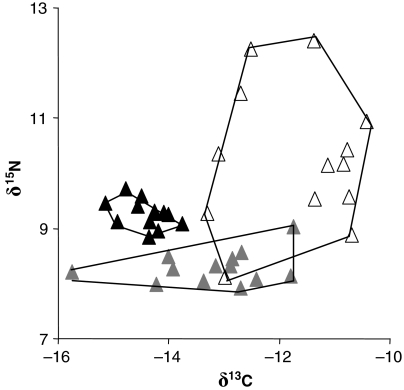
Each symbol represents an individual snapper and the lines represent the convex hull area used as a measure of niche width. White triangles are individuals from an unfragmented site, grey triangles from a partially fragmented site, and black triangles from a highly fragmented site (Cross Harbour, Sucking Fish, and Marsh Harbour, respectively, in Table 1).

Mean size of grey snapper individuals collected across all study systems was 164 mm (SD, ± 27). There was no relationship between degree of fragmentation and mean size of individuals from a site (*R*^2^ = 0.004, *P* = 0.85). To illustrate calculation of the TA metric, we depict individuals from three grey snapper populations in Fig. 1. The area which is outlined for each of the three populations is TA (the measure of trophic niche width). Because of contraction along both δ^13^C and δ^15^N axes, TA of the grey snapper populations in the most fragmented system is reduced 93% compared with grey snapper in an unfragmented system (0.6 compared with 8.9). The intraspecific ranges of both δ^13^C and δ^15^N are larger in the unfragmented system in this representative comparison.

**Table 1:** Number of potential prey taxa (classified at the familial level) collected in comprehensive sampling in the 11 creek systems

Decreasing fragmentation	Infaunal invertivore	Pelagic planktivore	Epibenthic microinvertivore	Macroinvertivore	Algivore/ detritivore	Scavenger	Molluscivore	Cleaner Shrimp	Familial richness	Functional group richness
Marsh Harbour	1	0	3	0	2	1	0	0	7	4
Double-Blocked	0	0	3	0	2	1	0	0	6	3
Indian River	1	1	3	0	2	0	0	0	7	4
Lake City	1	1	2	0	2	1	0	0	7	5
Sucking Fish	1	1	1	0	5	1	0	0	9	5
Triangle	1	1	1	0	5	1	1	0	10	6
Cherokee	1	1	3	1	3	1	1	0	11	7
Camp Abaco	1	1	2	0	4	1	1	0	10	6
Barracuda	1	1	3	1	7	1	1	1	16	8
Jungle	1	1	3	1	6	1	1	1	15	8
Cross Harbour	1	1	3	1	6	1	1	1	15	8

Creeks are oriented from most to least fragmented.

**Figure 2:**
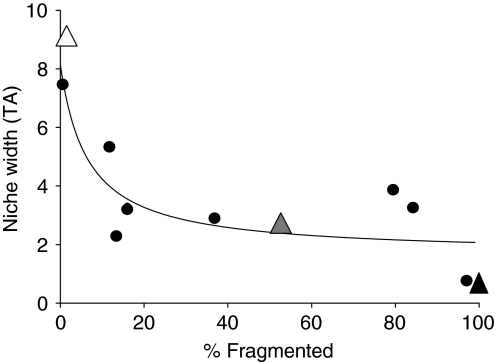
Niche width plotted as a function of percent fragmentation, with niche width estimated as convex hull area (TA) encompassing 13 individuals in each population. Each symbol represents the estimated niche width of a gray snapper population in one of 11 tidal creek systems, with triangles corresponding to the three sites depicted in Fig. 1.

Across the 11 study systems, percent fragmentation explained 80% of the variation in niche width of grey snapper (Fig. 2, best fit by a hyperbolic decay model, *R*^2^ = 0.80, *P* = 0.002). The collapse in niche width is likely the result of decreased prey diversity, measured as either the total number of prey families (Table 1, cubic model, *R*^2^ = 0.85, *P* = 0.003) or functional group classifications of those prey families (cubic model, *R*^2^ = 0.90, *P* = 0.001). Unfragmented systems support additional functional groups of prey on which snapper feed, including molluscivore crabs (e.g. mud crabs), pelagic planktivores (e.g. silversides) and epibenthic macroinvertivores (e.g. mantis shrimp). Functional redundancy also is lost in fragmented systems, especially through loss of primary consumers that rely on epiphytic algae and/or detrital resources as their primary food source.

**Figure 3:**
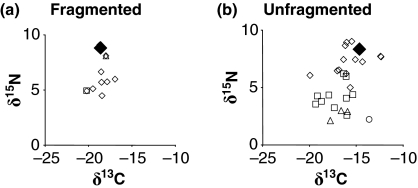
Stable isotope ratio data depicting resident taxa from food webs in one highly fragmented (Cross Harbour) and one unfragmented (Cherokee) tidal creek. Each point on the graph represents the mean value of 2–13 individuals of an individual species, with error bars around the mean omitted for simplicity. Diamonds = fish, squares = crustaceans, triangles = mollusks, and circles = other invertebrate taxa. The enlarged black diamond identifies the mean position of gray snapper in each food web.

Representative food webs, based on stable isotope ratios of all organisms resident to creeks, suggest fundamental differences in food web architecture between highly fragmented and unfragmented sites (Fig. 3). There is an overall decrease in taxonomic diversity (total number of symbols) and functional diversity (suggested by broader spacing in niche space) in fragmented systems (Layman *et al.* 2007). The latter is driven by larger ranges in both δ^13^C (7.5‰ compared with 3.3‰) and δ^15^N (6.9‰ compared with 4‰) in the unfragmented site. In each web, grey snapper have one of the highest δ^15^N values and intermediate δ^13^C values, which signify their position as a top predator (suggested by δ^15^N) that integrates energy flow pathways (suggested by δ^13^C) from prey with diverse isotopic signatures.

## Discussion

We demonstrate collapse of niche width in a generalist predator because of ecosystem fragmentation using a novel metric based on stable isotope ratios. It has been proposed that stable isotopes may provide a robust measure of niche width (Bearhop *et al.* 2004; Layman *et al.* 2007), and we provide the first quantitative measure to this end. Because the measure reflects relative variation both in trophic position and diversity of resource use, it may be a powerful approach in the study of both intraspecific and community-wide trophic structure. In the present study, niche width (i.e. TA) of grey snapper populations is significantly smaller in fragmented ecosystems because of contraction along both δ^13^C and δ^15^N niche dimensions. We discuss the underlying ecological reasons that drive niche width collapse, and explore implications of this collapse for the stability of the food webs in fragmented habitats.

Niche width collapse tracks the decrease in potential prey diversity in fragmented systems measured by either taxonomic or functional classifications. This reduction in prey diversity is the direct and indirect result of four ecosystem-level effects induced by fragmentation. First, ecosystem fragmentation disrupts metapopulation dynamics of prey populations, thereby reducing the supply of larvae, juveniles and adults to upstream areas (Gonzalez *et al.* 1998; Pringle 2006). Second, overall ecosystem size (volume of water and/or surface area of inudated wetland) can be reduced because of shallower water depths and increased sedimentation that fragmentation induces. Third, variation in physico-chemical conditions increases, especially in temperature and salinity, because of a lack of tidal flushing (Valentine *et al.* 2007b). And fourth, partially due to each of the preceding three factors, there is an overall decline in abundance of structural flora (e.g. seagrasses and macroalgae) that provide important habitat for a diverse suite of estuarine and marine fauna (Adams *et al.* 2006; McCall & Rakocinski 2007). Isolating the relative individual contribution of each of these mechanisms with respect to particular impacts on food web structure is difficult, but one net result, i.e. an overall reduction in prey diversity, is obvious and significant.

Reductions in prey diversity in fragmented ecosystems contribute directly to niche width collapse of top predators because predator individuals are constrained in their ability to choose among potential prey items. As such, in fragmented ecosystems, there is less scope for either chance (e.g. patchy prey distribution) or specialization (Bolnick *et al.* 2003) to result in individuals that exploit different sub-sets of available prey taxa. Variation in resource use among individuals is reflected by greater intraspecific variability in isotopic signatures (e.g. Cross Harbour population in Fig.1). But when prey diversity is low, all top predator individuals (both conspecifics and other species) must exploit the same taxa, thereby collapsing intraspecific niche variation (Marsh Harbour population in Fig. 1). This phenomena may be widespread following ecosystem fragmentation, as well as following other anthropogenic impacts, because biodiversity loss typically follows such disturbances (Fahrig 2003).

In our example, collapse in niche width was a function of reduction in diversity of basal resources supporting the food webs (represented by variation in δ^13^C), as well as a reduction in trophic level variation (δ^15^N) among organisms in fragmented food webs. Whereas terrestrial fragmentation does not necessarily change underlying characteristics of interior portions of fragmented patches (Fahrig 2003), aquatic ecosystem fragmentation, by definition, intrinsically alters overall physico-chemical characteristics (Pringle 2001). The most well-acknowledged instance of this phenomena is in freshwater ecosystems where dams alter hydrologic connectivity and subsequently affect food web structure and ecosystem function in both up- and downstream areas (Poff *et al.* 1997). In the present example, fragmented estuarine tidal creeks have altered upstream physico-chemical conditions, increased sedimentation and altered nutrient cycling, with the result being a net decrease in basal resource diversity in fragmented ecosystems. With fewer primary consumer niches available in fragmented creeks (e.g. no seagrass epiphytes or macroalgae on which to feed), there is an overall reduction in primary consumer diversity and thus fewer potential prey for top predators. This phenomena is represented by the reduction in community-wide δ^13^C range in fragmented systems (Fig. 3a), and is reflected in the decreased niche width of the top predator species.

Niche width collapse also is related to decreased variability in δ^15^N within food webs of fragmented creeks. For example, in the two representative webs depicted in Fig. 3, the range of δ^15^N is 2.9‰ larger in the unfragmented system. According to assumed values of fractionation for trophic transfers within food webs (e.g. 3.4 or 2.5, respectively, Post 2002b; Layman *et al.* 2005), the difference in δ^15^N range approximates a full trophic level of variation. As such, there seems to be *c*. 1 additional trophic level in an unfragmented ecosystem. This observation is supported by functional classifications of prey items, as unfragmented systems support intermediate predators (e.g. pelagic planktivores) that are not available in fragmented systems. This pattern is consistent with the ‘insertion mechanism’ that can affect relative trophic position of top predators through the addition of an intermediate link in a food chain (Post *et al.* 2000b; Post 2002a). We extend this idea to suggest that the insertion mechanism may affect the trophic position of individuals *within* a population of conspecifics. That is, if particular individuals specialize (*sensu*Bolnick *et al.* 2003) on a prey item that is only found in unfragmented creeks, those individuals may have a higher trophic position within the web than is possible for conspecifics in a fragmented system. This partially drives the overall increase in population niche width in unfragmented systems.

Other studies have found that population niche width expands when resources become scarce or intraspecific competition otherwise increases (Wilson & Turelli 1986; Bolnick 2001; Svanbäck & Persson 2004). Such studies are typically conducted in systems with a variety of alternative resources among which consumers could choose, e.g., in lakes where fish can utilize distinct pools of benthic and limnetic resources (Svanbäck & Persson 2004). In such systems, consumers can switch to suboptimal resources when preferred prey become scarce. Yet in the instance considered herein, predators do not have a distinct alternative prey resource to exploit because diversity of prey decreases with fragmentation, as is reflected in the representative fragmented food web in Fig. 3. When anthropogenic impacts significantly reduce available resource pools, population niche width expansion cannot ensue in response to resource scarcity because prey choice is so constrained.

**Figure 4:**
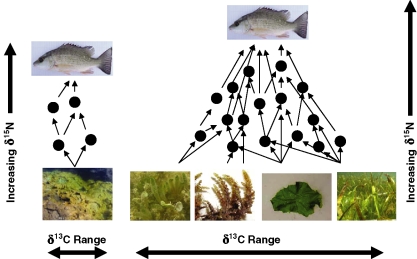
Conceptual depictions of the ‘sink’ food webs (i.e. all energy flow pathways that culminate in grey snapper) in a typical fragmented and unfragmented creek system, as based on > 3500 individual isotope samples, hundreds of stomach content analyses, and quantitative floral and faunal surveys. Identity of intermediate consumers are not given to provide for a more generalized view of food web architecture.

Based on > 3500 individual isotope samples, hundreds of stomach content analyses, and extensive quantitative floral and faunal surveys, we are able to depict conceptualized energy flow pathways in typical fragmented and unfragmented creek systems (Fig. 4). Three critical observations can be derived from these conceptual trophic architectures. First, generalist top predators (grey snapper, as well as other species such as barracuda, needlefish and tarpon) have intermediate positions along a δ^13^C niche axis, as they integrate across multiple energy flow pathways (Vander Zanden & Vadeboncoeur 2002; Rooney *et al.* 2006). By feeding on a diverse suite of prey with variable trophic roles, their resulting position along the δ^13^C niche axis is intermediate relative to other taxa within the web. Second, a more narrow range of consumer values along the δ^13^C axis in the fragmented systems is a direct function of a single basal resource pool (microalgal mat) dominating the substrate. This pool serves as the ultimate source of carbon for all consumer species. Third, in unfragmented systems, hundreds (and perhaps thousands) of unique energy flow pathways connect the base of the food web to top predators, because of the increased diversity of basal resource pools, greater abundance of intermediate prey taxa, and increased incidence of omnivory. In fragmented systems, there is a drastic overall simplification of energy flow pathways and web architecture.

The importance of numerous, heterogeneous, energy flow pathways has been implicated as a primary mechanism stabilizing food webs (McCann *et al.* 1998; Post *et al.* 2000a; Rooney *et al.* 2006). Energy flow ‘asymmetry’ occurs when top predators utilize multiple pathways that differ in productivity and turnover rate, thereby coupling energy flows that cycle at different temporal scales and providing stability to food webs (Rooney *et al.* 2006). Niche width collapse is a direct indication that such asymmetry is lost in fragmented habitats. In fragmented systems, pathways of energy flow are constrained to originate with a single basal resource pool and pass through fewer intermediate consumers. The end result is an overall homogenization in energy flow pathways and ultimately a less stable food web structure (*sensu*Rooney *et al.* 2006).

Even though many generalist species can persist in highly fragmented ecosystems, ecological roles of these species may be altered significantly. Niche width collapse of the generalist species results from an overall simplification of food web structure which may, in turn, render the top predators more susceptible to population fluctuations and more likely to face extinction through time (Tilman *et al.* 1994). Because alteration in the role of top predators can have cascading effects throughout entire food webs (Terborgh *et al.* 2001), collapse in niche width ultimately can have fundamental effects on ecosystem function. With continuing erosion of local and global biodiversity, ecologists should continue to move beyond documenting the presence/absence of species in altered ecosystems, and instead work to better understand how anthropogenic impacts intrinsically alter ecological roles of species.
